# Cytokine profiling reveals HLA-linked Th2 and Th17 driven immune activation in pemphigus vulgaris patients and genetically susceptible healthy controls

**DOI:** 10.3389/fimmu.2024.1500231

**Published:** 2024-12-04

**Authors:** Rebekah R. Schwartz, Kristina Seiffert-Sinha, Animesh A. Sinha

**Affiliations:** Department of Dermatology, University at Buffalo Jacobs School of Medicine and Biomedical Sciences, Buffalo, NY, United States

**Keywords:** Pemphigus, HLA, cytokine, autoimmunity, Th17, Th2

## Abstract

**Introduction:**

Cytokines and chemokines direct the inflammatory response and may serve as markers of immune dysregulation in Pemphigus vulgaris (PV), an autoimmune blistering skin disorder. Previous studies on limited numbers of patients and cytokine profiles in PV have produced equivocal results regarding the role these mediators play in disease.

**Methods:**

In this study, we interrogated serum samples from 116 PV patients and 29 healthy controls by multiplexed bead array assays across a comprehensive set of cytokines and chemokines covering several functional categories, including IL-1α, IL-1β, IL-2, IL-4, IL-5, IL-6, IL-8, IL-9, IL-10, IL-12, IL-13, IL-15, IL-17, IL-21, IL-22, IL-23, TNFα, IFNγ, MCP-1, and Eotaxin.

**Results:**

We found that patients with PV generally display an activated cytokine and chemokine immune response compared to controls, but also show remarkable interindividual heterogeneity in terms of cytokine levels, with a limited activation of different T helper cell pathways in different patients. Surprisingly, we also found that healthy individuals that carry the PV susceptibility alleles HLA DR4 (DRB1*0402) and/or DR6 (DQB1*0503) (HLA-matched controls) show an upregulation of cytokine and chemokine levels that are on par with those seen in PV patients for certain pro-inflammatory, Th2, and Th17 mediators and IL-8, while healthy controls that did not carry the PV susceptibility alleles (HLA-unmatched controls) express significantly lower levels of these cytokines and chemokines.

**Discussion:**

Our data suggest the existence of a limited immune activation linked to the presence of key PV associated HLA alleles regardless of disease status. Interestingly, the cytokines IL-10 and IL-15 were found to be significantly downregulated in the HLA-matched control group, suggesting the presence of a possible counter-regulatory function in genetically susceptible but disease-free individuals.

## 1 Introduction

Pemphigus vulgaris (PV) is a potentially life-threatening autoimmune blistering skin disorder characterized by a well-defined humoral response directed against specific desmosomal proteins involved in cell-cell adhesion and maintenance of epidermal integrity. The hallmark for autoimmunity in PV is the presence of autoantibodies specific for desmoglein (Dsg)-3, and in some cases Dsg-1, both in serum and in lesional skin. The “desmoglein compensation hypothesis” posits that lesion morphology (mucosal *vs*. mucocutaneous) can be explained by autoantibody patterns (anti-Dsg3 only *vs*. a combination of anti-Dsg3 and anti-Dsg1, respectively) (1), however, the validity of this theory has been challenged recently (2). While the target organ damage is ultimately mediated by autoantibodies, it is generally accepted that B cell activation requires the participation of T helper cells necessary for immunoglobulin (Ig) production and class switching. T cell recognition of Dsg3 and/or Dsg1 epitopes appears to be the first step in the disease initiation, leading to B-cell activation and production of Dsg3/1 specific immunoreactants (3–6).

The importance of T cell involvement in disease pathogenesis is underscored by the strong genetic association of PV to the HLA DR4 (DRB1*0402) and DR6 (DQB1*0503) haplotypes (7, 8). T cells play a role in the inception and maturation of the humoral immune response, thus bridging the association of PV with MHC and generation of autoantibodies (9). Of course, these haplotypes are also found in healthy individuals. In fact, the vast majority of healthy individuals who express HLA DR4 (DRB1*0402) or DR6 (DQB1*0503) gene sequences do not progress to a disease state. Why healthy individuals who carry these disease risk elements remain healthy remains a mystery.

While the coordination and regulation of the autoimmune response in PV has not been fully elucidated, cytokines and chemokines are likely to orchestrate the interplay between cellular and humoral responses. Cytokines act as mediators for disease activation and maintenance in autoimmune disorders, but also operate as initiators of disease remission and induction of the tolerogenic state (10). Produced by, and acting on various cellular components of the immune cascade, they participate in both effector and regulatory pathways (10).

Limited attempts have been made to define cytokine and chemokine expression patterns in PV. Apart from a recent publication (11), previous studies typically examined only a handful of cytokines in small sample sizes using disparate methods to quantify cytokine concentrations. Thus, perhaps unsurprisingly, reported results are equivocal. In fact, many cytokines in PV were found to be upregulated in one study, only to be shown to have no significant change or even to be downregulated in another (3, 12–59). **Table 1** shows a comprehensive literature review of previous serum cytokine studies, particularly which studies show an elevation, decrease, or no significant change in PV patients versus healthy controls, as well as how this data compares to new findings from this study that will be discussed in detail below. Across studies, heterogeneity amongst patients and control subjects in terms of disease phase, clinical phenotype, gender, HLA-association, and treatment may have contributed to ambivalent results, factors that are particularly difficult to account for in small sample sizes.

**Table 1:** Review of literature findings regarding serum cytokine levels in PV patients compared to controls.

Cytokine	Number of Studies Showing Increased in PV	Number of Studies Showing No Change in PV	Number of Studies Showing Decreased in PV	Our Findings		
				Inc in PV vs. all	Inc in PV vs. MCR	Inc in PV vs. UMCR
CCL-11	-	-	1 (27)	Not analyzed		
Eotaxin	-	1 (11)	-	No change		
IFNα	-	1 (11)	-	Not analyzed		
IFNγ	7 (11, 20, 24, 26, 27, 29–31)	4 (13, 21, 37, 41)	3 (14, 25, 38)	No change		
IL-1α	7 (18, 32–37)	4 (11, 13, 19, 43)	-			X
IL-1β	8 (11, 16, 32–37)	4 (13, 19, 27, 43)	1 (22)			X
IL-2	3 (11, 16, 24)	6 (13, 19, 21, 27, 41, 44)	3 (25, 31, 38)	X	X	X
IL-4	8 (14, 24, 25, 29, 38–42)	6 (11, 13, 21, 27, 44, 55)	1 (31)	X		
IL-5	1 (11)	5 (13, 16, 21, 24, 27)	-	X	X	X
IL-6	10 (13, 21, 22, 26, 30, 36–38, 43, 44)	8 (11, 16, 19, 20, 41, 51, 56, 57)	-			X
IL-7	1 (16)	1 (13)	-	Not analyzed		
IL-8	3 (16, 20, 26)	3 (11, 13, 27)	-			X
IL-9	-	2 (11, 27)	-	X	X	X
IL-10	9 (11, 19, 25, 30, 39–42, 45)	5 (13, 18, 20, 21, 27)	1 (31)	X	X	
IL-12	3 (11, 21, 30)	3 (13, 27, 37)	-	No change		
IL-13	1 (11)	1 (24)	-			X
IL-15	1 (46)	1 (11)	-	X	X	
IL-17	5 (17, 19, 27, 30, 47)	1 (21)	1 (56)	No change		
IL-17A	3 (11, 12, 48)	2 (16, 20)	-	No change		
IL-18	1 (11)	-	-	Not analyzed		
IL-21	1 (49, 59)	2 (11, 16)	1 (55)			X
IL-22	1 (11)	-	1 (22)	X	X	X
IL-23	2 (27, 50)	2 (11, 19)	1 (56)	X		X
IL-27	1 (51)	-	-	Not analyzed		
IL-31	-	-	1 (11)	Not analyzed		
IL-33	1 (52)	-	-	Not analyzed		
IL-36	1 (48)	-	-	Not analyzed		
IP-10	-	-	2 (11, 27)	Not analyzed		
MCP-1	-	1 (11)	1	No change		
TGF-β	2 (12, 18)	4 (13, 17, 27, 41)	3 (30, 56, 58)	Not analyzed		
TNFα	13 (11, 13, 18, 23, 32, 33, 35–37, 44, 51, 53, 54)	3 (19, 20, 43)	-			X
TNFβ	-	1 (11)	-	Not analyzed		

To overcome these challenges, we conducted an extensive analysis of serum cytokines/chemokines in PV, both in terms of the range of soluble mediators studied and the number of patient and control samples analyzed. We comprehensively compared multiple serum cytokines in defined clinical categories of PV. Unlike previous studies, we further explored the contribution of HLA haplotype to the cytokine milieu in both patients and controls to pinpoint the role of cytokines in genetically susceptible, but disease-free subjects. We utilized a multiplex bead array platform to evaluate the expression of 20 cytokines and chemokines categorized by function: 1) pro-inflammatory (IL-1α, IL-1β, IL-6, and TNFα), 2) Th1 (IFNγ, IL-2, IL-12), 3) Th2 (IL-4, IL-5, IL-13), 4) Th9 (IL-9), 5) Th17/Th22 (IL-17, IL-21, IL-22, IL-23), 6) Treg (IL-10), 7) NK cell (IL-15), and 8) chemokines (IL-8, MCP-1, Eotaxin) in 130 serum samples obtained from 116 PV patients in different phases of disease activity and remission, along with 15 healthy control subjects that carried the PV-associated HLA alleles DRB1*0402 and/or DQB1*0503 (termed here as HLA-matched controls) and 14 healthy control subjects that did not carry the known PV-associated HLA alleles (HLA-unmatched controls).

## 2 Materials and methods

### 2.1 Patient population and demographics

The patients included in this study were recruited from the Dermatology outpatient clinics at Weill-Cornell Medical College (IRB 0998-398), Michigan State University (IRB 05-1034), and the University at Buffalo (IRB 456887), and annual meetings of International Pemphigus and Pemphigoid Foundation (IPPF). Institutional review boards at each of the participating institutions reviewed and approved this study.

After obtaining written informed consent, researchers acquired detailed demographic and clinical information from patients and control subjects. Subsequently, venous blood samples were drawn and each patient’s serum was separated by centrifugation and stored at minus 80°C until use.

The diagnosis of PV was based on established clinical, histopathologic, and/or serologic criteria. Healthy controls did not have a diagnosis or history of PV. All study procedures were identical between healthy controls and PV patients. We included 130 serum samples from 116 patients in different phases of disease (active and remittent) and varying degrees of therapy and 29 samples from 29 control subjects, of which 15 were HLA-matched controls (i.e. carriers of the HLA-susceptibility alleles DRB1*0402 and/or DQB1*0503) [MCR] and 14 were HLA-unmatched controls (i.e. non-carriers of PV susceptibility HLA alleles) [UMCR]. The demographic data for our study population is summarized in **Table 2**.

**Table 2:** Demographic data and HLA association.

	PV patients	HLA-matched Controls	HLA-Unmatched Controls
Total number of samples	130	15	14
Total number of subjects	116	15	14
Average Age in years [range]	56 [17-85]	51 [22-81]	51 [28-77]***
Female (n)	96	10	8
Male (n)	34	5	6
Female: Male ratio	2.82	2.00	1.33
Active	55	N/A	N/A
Remission	75	N/A	N/A
HLA positive*	98	15	0
HLA negative**	12	0	14
Active Disease	55	N/A	N/A
Remission	75	N/A	N/A
Mucosal Phenotype	48****	N/A	N/A
Mucocutaneous Phenotype	75****	N/A	N/A
Off Therapy	40	N/A	N/A
Minimal Therapy	34	N/A	N/A
More than Minimal Therapy	56	N/A	N/A

*HLA-positive refers to subjects who were positive for the PV associated haplotypes DRB1*0402 or DQB1*0503. **HLA-negative refers to subjects who were negative for the PV associated haplotypes. *** One of the HLA-unmatched controls did not provide a date of birth; this number excludes that control. **** 3 patients had cutaneous only disease and 4 had unclear phenotype.

We used consensus guidelines developed by the International Pemphigus Committee (60) to determine disease activity in PV patients. Briefly, patients were considered to be active if they had 3 or more non-transient lesions (lasting more than one week) and/or extension of existing lesions. Patients were considered to be remittent if they had no new or established lesions for at least 2 months.

We assigned a therapy status to each patient based on consensus guidelines (60). Patients receiving > 10mg/day of prednisone, IVIg, cyclosporine, dapsone, rituximab, other biologic agents, and/or other adjuvant therapies at full therapeutic doses were defined as “more than minimal” therapy. “Minimal” therapy was defined by prednisone doses of ≤ 10mg/day and/or minimal adjuvant therapy as defined in (60) for at least 2 months. “Off” therapy was reserved for patients that were not receiving any systemic therapy.

### 2.2 HLA-typing

High resolution HLA-typing of patients and controls was done at Rogosin Institute (New York, NY) and the Tissue Typing Laboratory at Michigan State University (East Lansing, MI). HLA Class II alleles, DRB1 and DQB1, were amplified using sequence specific primers as previously described (61). Patients and controls that expressed the HLA associated alleles (homozygous or heterozygous) were classified as “HLA-positive” or HLA+. Those without were classified as “HLA-negative” or HLA-.

### 2.3 Multiplex bead assay

Patient and control serum samples were distributed amongst two panels of customized Millipore Multiplex 96-well plates (EMD Millipore, Saint Charles, MO). The first panel included: IL-1α, IL-1β, IL-2, IL-4, IL-5, IL-6, IL-8, IL-9, IL-10, IL-12, IL-13, IL-15, TNFα, IFNγ, MCP-1, and Eotaxin, and the second panel included the Th17 cytokines IL-17, IL-21, IL-22, and IL-23. An aliquot of 50 µl from each PV patient or control serum was added to each well aside internal control samples of known concentrations. Samples were run in duplicates. Standards were serially diluted 1:3 to generate a nine-point standard curve. Analyte capture was carried out according to manufacturer’s instructions. Cytokine concentrations (pg/mL) were measured using the Luminex 200 system with xPONENT version 3.1 software and assessed using BeadView Analysis software. Raw values are compared to standard curves for each cytokine. Cytokines are considered 0 or undetectable when falling within the lower nonlinear portion of the standard curve. We report raw values (pg/mL) as our standard curves do not provide cutoffs for positivity. In addition, we used unbiased hierarchical clustering using Euclidean distance with Morpheus software (https://software.broadinstitute.org/morpheus) to identify any grouping or patterns in cytokine levels.

### 2.4 Statistical analysis

For certain analyses, PV samples were subclassified according to major clinical parameters of interest and further placed in subgroups established by clinical criteria (in parentheses), i.e. disease phase (active or remission), therapy status (off or minimal or more than minimal), clinical phenotype (mucosal or mucocutaneous), and gender (male or female). Control patients were separated into HLA-matched and HLA-unmatched groups.

Statistical analysis was conducted between clinical subgroups including PV *vs*. all controls, HLA-matched controls *vs*. HLA-unmatched controls, and within the PV group between disease activity (active *vs*. remittent), gender (male *vs*. female), morphology (mucocutaneous *vs*. mucosal), and therapy (off therapy *vs*. minimal therapy, minimal therapy *vs*. greater than minimal therapy, and greater than minimal therapy *vs*. off therapy).

Groups were compared by a heteroscedastic T-test. A p-value ≤ 0.05 indicated that the difference between the means, μ1 and μ2, of the populations from which data1 and data2, respectively, were sampled is significantly differently from Δμ=0. The null hypothesis, Δμ=0, was rejected when p-value < 0.05.

## 3 Results

### 3.1 Cytokines in PV patients show wide variability and heterogeneity, but are pathway specific and generally elevated compared to healthy controls

**Figure 1:**
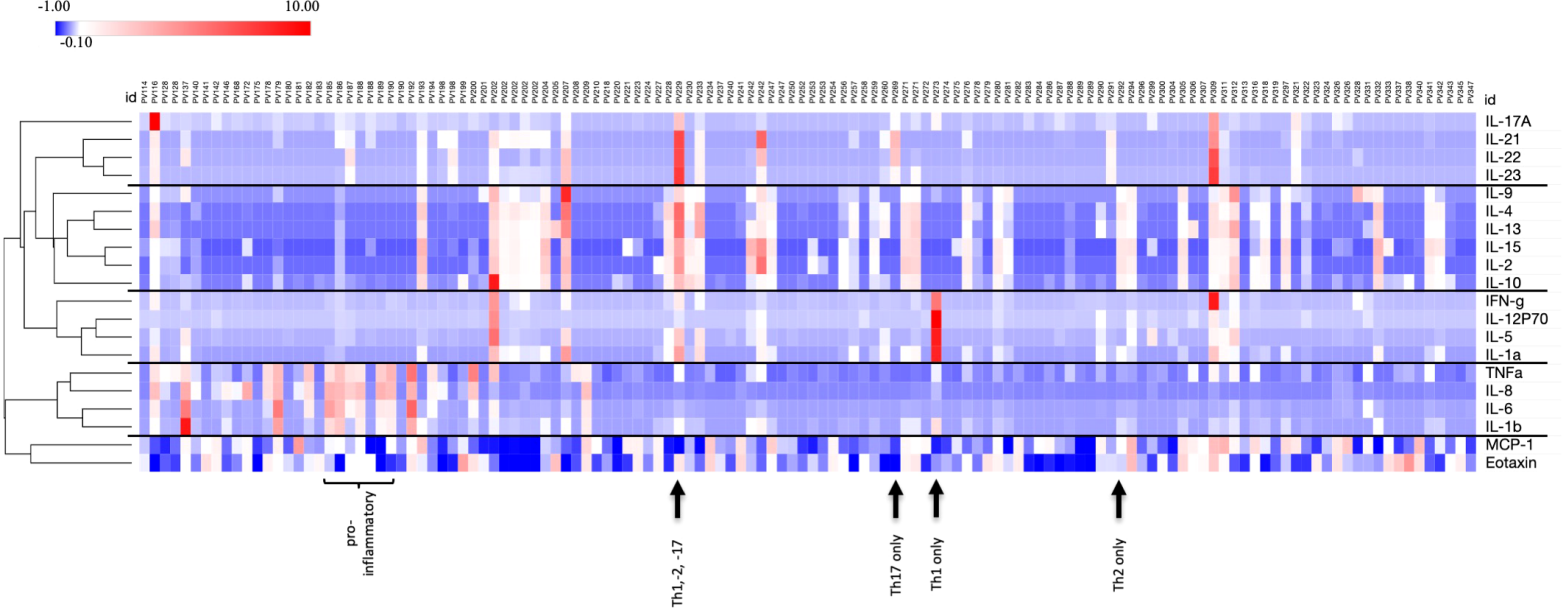
Wide variation and patient specificity for cytokine profiles. Z-score transformed data in heatmap format shows all cytokine concentrations for each PV patient (n=130) simultaneously. Unbiased clustering identifies cytokine subgroups with similar expression levels for individual patients. Examples for a certain cytokine profile are identified as Th2 only (PV292), Th1 only (PV273), Th17 only (PV269), combined Th1, -2, 17 (PV229) and proinflammatory pattern (PV185 to 190). Heatmap was created using Morpheus Software. https://software.broadinstitute.org/morpheus.

We examined 20 cytokines/chemokines in 116 PV patients and 29 healthy controls. For each of the cytokines analyzed, we observed large interindividual differences among serum concentrations ranging from 0 pg/mL (undetectable) to high values such as ~73,000 pg/mL for IL-23, for example. This wide variation was particularly pronounced in the patient population. Interestingly, we saw a remarkable heterogeneity among patients in terms of which cytokines were elevated and also observed that among a given patient cytokine values were typically elevated across just one or two specific pathways, but not for all cytokines analyzed. In order to identify any patterns of cytokine responses (i.e. whether cytokines are regulated in groups), we used an unbiased hierarchical clustering approach. This unbiased clustering approach identified 5 clusters of cytokine responses: (i) Th17: IL17A, IL-21, IL-22, IL-23; (ii) Th2-dominant: IL-2, IL-4, IL-9, IL-10, IL-13, IL-15; (iii) Th1-dominant: IL-1α, IL-2, IFNγ, IL-5; (iv) generally proinflammatory: IL-1β, IL6, IL-8 and TNFα; and (v) the chemokines MCP-1 and eotaxin (**Figure 1**). Some patients showed an elevation of one or two T helper cell pathways, while others had high levels of proinflammatory cytokines in the absence of T helper pathway cytokines. Conversely, we observed a sizeable number of PV patients that had undetectable levels for the majority of cytokines, suggesting that cytokine responses are remarkably patient-specific and not universally present in all.

Despite this variation among patients, all of the cytokines and chemokines analyzed had higher mean levels in PV patients compared to all healthy controls (**Table 3A**), with IL-2, IL-4, IL-5, IL-9, IL-10, IL-15, IL-22, and IL-23 reaching statistical significance (p-value <0.05) (**Table 3B**), indicating a general immune activation in patients compared to controls.

**Table 3:** Cytokine/Chemokine levels and group comparison in PV patients and healthy controls.

A					
		Mean (± SD) (pg/mL) Cytokine/Chemokine Level of Patients & Control Groups			
		PV (n=130)	ALL Controls (n=29)	HLA-Matched Controls (n = 15)	HLA-Unmatched Controls (n=14)
Pro - Inflammatory	IL-1α	76.60 (246.4)	27.60 (71.8)	41.35 (96.6)	12.86 (24.5)
	IL-1β	20.75 (69.1)	14.69 (48.1)	26.03 (65.6)	2.54 (5.2)
	IL-6	86.49 (284.3)	61.40 (218.4)	115.8 (298.0)	3.11 (5.2)
	TNF-α	30.32 (48.9)	28.47 (62.9)	45.50 (85.0)	10.22 (5.8)
Th1	IFN-γ	63.01 (319.7)	18.26 (36.8)	24.06 (38.6)	12.04 (35.1)
	IL-2	4.95 (9.4)	1.64 (3.5)	1.27 (2.9)	2.04 (4.1)
	IL-12	61.53 (416.7)	8.21 (30.7)	12.62 (42.4)	3.50 (7.1)
Th2	IL-4	32.52 (67.6)	16.04 (27.5)	15.38 (28.2)	16.75 (27.8)
	IL-5	5.26 (22.0)	0.63 (2.0)	1.22 (2.8)	0.00 (0.0)
	IL-13	17.51 (36.2)	13.20 (33.3)	18.71 (44.3)	7.31 (14.1)
Th9	IL-9	3.43 (9.2)	0.68 (1.7)	0.51 (1.4)	0.87 (2.0)
Th17	IL-17	5.55 (28.1)	4.28 (10.5)	6.11 (13.2)	2.32 (6.4)
	IL-21	4.13 (14.9)	1.76 (3.6)	2.85 (4.6)	0.59 (1.3)
	IL-22	105.31 (425.3)	16.15 (45.8)	25.37 (59.2)	6.28 (23.5)
	IL-23	1972.93 (9348.6)	220.19 (539.8)	346.00 (706.2)	85.39 (229.8)
Chemokine	IL-8	655.03 (1561.2)	625.31 (1893.9)	1076.38 (2587.9)	142.03 (162.8)
	Eotaxin	201.74 (108.6)	174.86 (70.9)	160.39 (76.2)	190.37 (63.9)
	MCP-1	841.06 (499.2)	839.21 (471.4)	894.13 (583.5)	780.36 (323.7)
NK	IL-15	6.65 (11.1)	2.54 (5.0)	1.99 (3.8)	3.13 (6.2)
Regulatory	IL-10	17.32 (38.0)	7.44 (17.2)	3.89 (6.7)	11.25 (23.7)

B					
		P-values for Group Comparisons			
		PV *vs*. ALL Controls	PV *vs*. HLA-Matched Controls	PV *vs*. HLA-Unmatched Controls	Matched Controls *vs*. Unmatched Controls
Pro - Inflammatory	IL-1α	0.056	0.292	0.005**	0.286
	IL-1β	0.53	0.773	0.004**	0.189
	IL-6	0.600	0.722	0.001**	0.165
	TNF-α	0.883	0.511	0.00002***	0.131
Th1	IFN-γ	0.123	0.193	0.087	0.387
	IL-2	0.002**^3^	0.002**	0.042*	0.572
	IL-12	0.152	0.202	0.115	0.425
Th2	IL-4	0.037*	0.076	0.106	0.895
	IL-5	0.020*	0.052(*)	0.007**	0.108
	IL-13	0.539	0.921	0.046*	0.358
Th9	IL-9	0.002**	0.001**	0.009**	0.580
Th17	IL-17	0.687	0.894	0.285	0.331
	IL-21	0.107	0.470	0.010*	0.087
	IL-22	0.021*	0.049*	0.010*	0.262
	IL-23	0.036*	0.055	0.023*	0.193
Chemokine	IL-8	0.938	0.546	0.0005***	0.184
	Eotaxin	0.103	0.072	0.567	0.260
	MCP-1	0.985	0.739	0.538	0.519
NK	IL-15	0.003**	0.001**	0.079	0.558
Regulatory	IL-10	0.035*	0.0005***	0.406	0.280

* indicates significant value: (*) 0.05< p <0.1, * p<0.05, ** p<0.01, ***p<0.001.

### 3.2 Healthy controls carrying PV-associated HLA susceptibility alleles exhibit a heightened cytokine activity within pro-inflammatory, Th2-, Th17-, and IL-8 pathways

We noticed some heterogeneity regarding cytokine levels within the control population similar to what we had observed in the patient population. To investigate whether cytokines differed based on HLA association, we divided the control population into those that expressed the known PV-associated HLA-alleles DRB1*0402 and DQB1*0503 (“HLA-matched controls”, n=15) and those that did not express any of the known PV associated HLA alleles (“HLA-unmatched controls”, n=14) and then analyzed which cytokines differentiated PV from both control groups or either one of the control groups alone.

**Figure 2:**
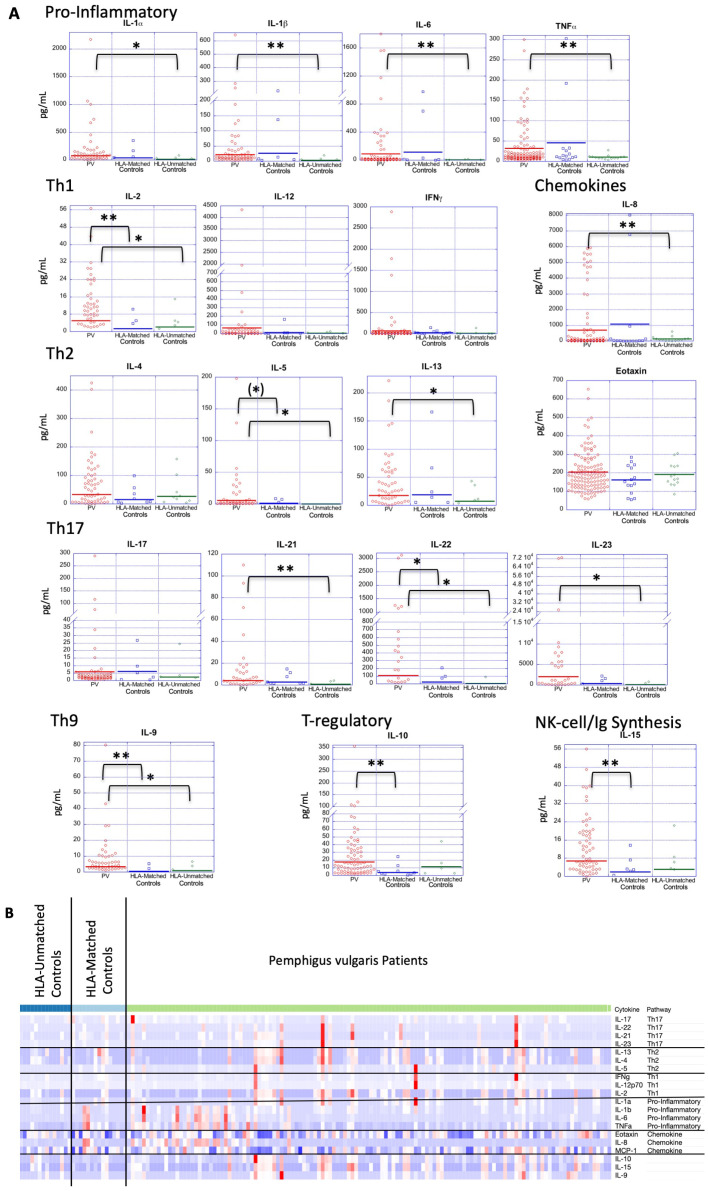
Cytokine expression profiles in PV patients and controls, both HLA-matched and HLA-unmatched. **(A)** Dot plots depict cytokine levels in PV patients and controls (HLA-matched and HLA-unmatched), with each dot representing an individual sample. The horizontal bar in each category represents the mean. P-values ≤ 0.05 are indicated with *; p-values ≤ 0.005 are indicated with **. **(B)** Z-score transformed data in heatmap format shows all cytokine concentrations for each PV patient (n=130) an HLA-matched (n=15) and HLA-unmatched controls (n=14) simultaneously. Cytokines are listed in order of functional subgroup. Heatmap was created using Morpheus Software. https://software.broadinstitute.org/morpheus.

We observed that the higher serum levels of multiple cytokines persisted in a more limited set of cytokines when compared to HLA-matched or HLA-unmatched control groups separately. Of the cytokines that were significantly elevated in PV compared to all controls, only IL-2, IL-9, and IL-22 displayed significantly higher mean levels than both HLA-matched controls or HLA-unmatched controls. IL-5, though not significant, was highly trending towards significance (**Figure 2A** and **Tables 3A, B**).

Another group of cytokines retained or achieved significance only when patients were compared to “HLA-unmatched” healthy controls, i.e. those that do not carry PV-associated genetic susceptibility elements. PV patients had significantly elevated levels of the Th2 cytokine IL-13, the pro-inflammatory cytokines IL-1α, IL-1β, IL-6, TNFα and the chemokine IL-8 in this comparison (**Figure 2A** and **Tables 3A, B**). No significant differences were found between PV patients and “HLA-matched” controls for these cytokines. “HLA-unmatched” controls expressed very low levels of several cytokines (IL-1α: 12.01 ± 23.9 pg/mL, IL-1β: 2.55 ± 5.2 pg/mL, IL-6: 3.12 ± 5.2 pg/mL, TNFα: 10.22 ± 5.8 pg/mL, IL-8: 142.21 ± 162.9 pg/mL, and IL-13: 7.31 ± 14.1 pg/mL, respectively), while “HLA-matched” controls expressed levels on par or greater than those found in PV patients for numerous cytokines (IL-1α: 41.35 ± 96.6 pg/mL, IL-1β: 26.03 ± 65.7 pg/mL, IL-6: 115.8 ± 297.9 pg/mL, TNFα: 45.51 ± 85.0 pg/mL, IL-8: 1076.47 ± 2587.9 pg/mL, and IL-13: 18.7 ± 44.4 pg/mL, respectively) (**Figure 2A** and **Tables 3A, B**).

These findings are also reflected when looking at cytokine concentrations in a heatmap format, with highest expression found in PV patients, followed by HLA-matched controls, with visibly lower values for HLA-unmatched controls (**Figure 2B**). Our data indicate that healthy controls that carry PV-associated HLA alleles exhibit a limited immunological activation similar to PV patients in terms of the pro-inflammatory cytokines IL-1α, IL-1β, IL-6, TNF-α, the Th2 cytokine IL-13, the Th17 cytokines IL-21 and IL-23, and the chemokine IL-8.

Interestingly, we observed that mean IL-10 and IL-15 concentrations were lower in both the “HLA-matched” and “HLA-unmatched” control groups than the levels found in PV patients (**Figure 2A**). However, mean IL-10 and IL-15 levels in “HLA-matched” controls (means: 3.89 ± 3.8 pg/mL and 1.99 ± 6.7 pg/mL, respectively) were found to be even lower than that seen in “HLA-unmatched” controls (means: 11.25 ± 6.2 pg/mL and 3.13 ±23.7 pg/mL, respectively) (**Table 3A**). In fact, when compared to PV patients, IL-10 and IL-15 cytokine levels were significantly lower only in “HLA-matched” controls (p-value: 0.0007 and 0.001, respectively) but not in unmatched controls (**Figure 2** and **Table 3B**). The differences noted above raise the intriguing possibility that the downregulation of these two cytokines may contribute to mechanisms that protect genetically susceptible individuals from developing Pemphigus vulgaris.

### 3.3 PV patients do not differ in cytokine expression based on their level of disease activity, apart from a limited number of pro-inflammatory cytokines which are elevated in remission

**Figure 3:**
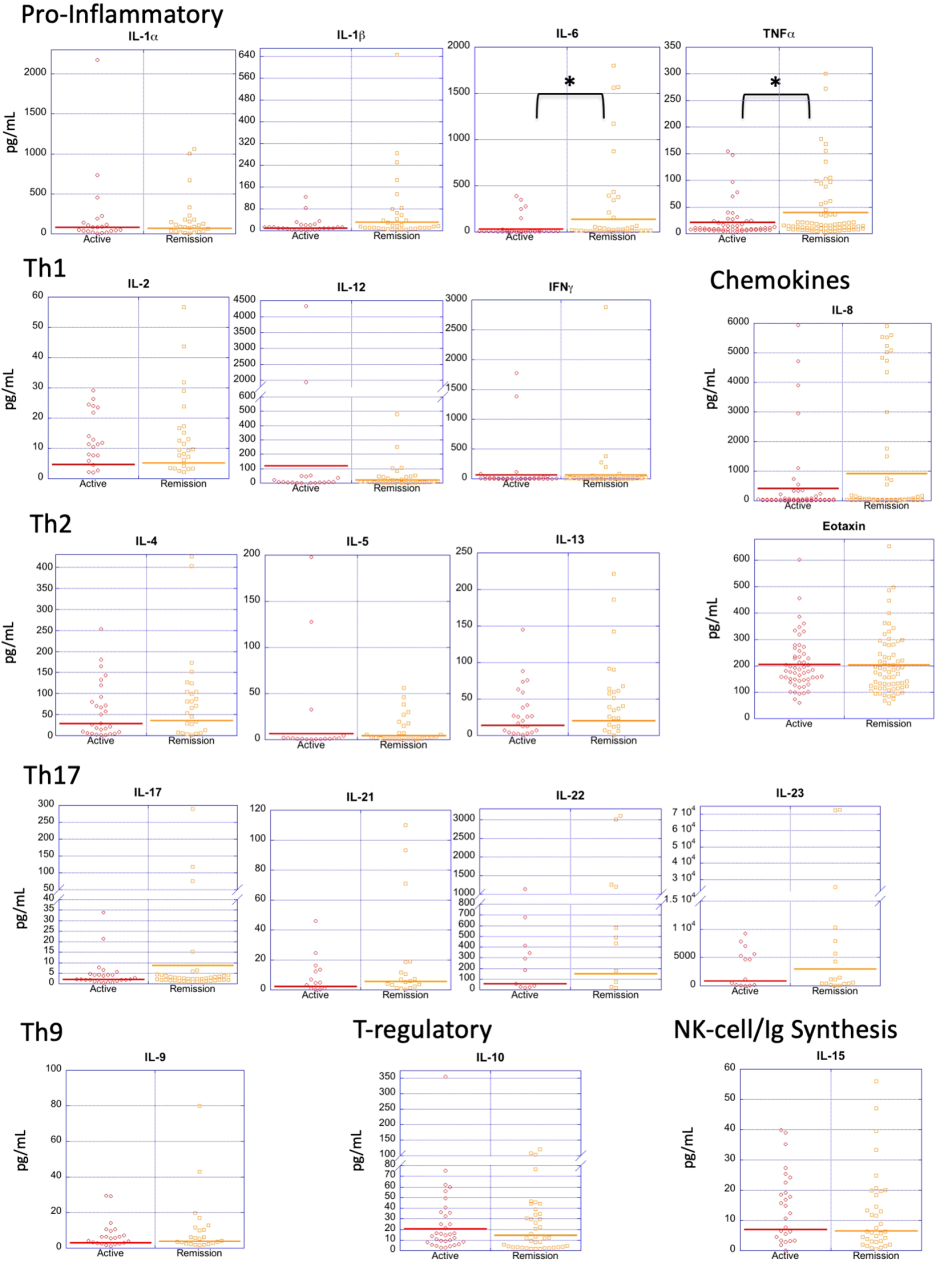
Cytokine expression profiles by disease activity. Dot plots depict cytokine levels in PV patients in active disease and remission, with each dot representing an individual sample. Only IL-6 and TNF-α were significantly different between patients with active PV *vs*. remittent PV. The horizontal bar in each category represents the mean. P-values ≤ 0.05 are indicated with *.

In order to assess the extent to which the level of disease activity influences serum cytokine levels in PV, we divided patients into those who were in active disease (n=55) *vs* remission (n=75) (**Figure 3**). Surprisingly, we found no significant differences for most of the cytokines analyzed, with both the active and remittent groups continuing to show remarkable heterogeneity of cytokine levels. Even more surprisingly, TNFα and IL-6 were significantly higher in remission versus active disease and the pro-inflammatory cytokine IL-1β and chemokine IL-8 had increased expression in remission *vs*. active disease which trended toward significance (**Figure 3** and **Table 4**). These data indicate that active disease is not associated with an across-the-board activated cytokine profile. If anything, a limited number of pro-inflammatory cytokines increase as the disease goes into remission. One speculation for this surprising finding is that a persistent proinflammatory milieu does not preclude or prevent the critical (and thus far unknown) factors responsible for limiting lesional activity.

**Table 4:** Cytokine/Chemokine levels and group comparison based on disease activity.

		PV active	PV remission	p-value
		Mean (SD)	Mean (SD)	
Pro-Inflammatory	IL-1α	85.40 (310.88)	72.04 (196.14)	0.782
	IL-1β	9.26 (20.80)	31.77 (92.63)	0.055
	IL-6	31.04 (84.43)	139.47 (379.32)	0.0249*^2^
	TNF-α	20.95 (31.41)	40.12 (59.91)	0.0246*
Th1	IFN-γ	66.18 (299.39)	64.98 (352.48)	0.983
	IL-2	4.75 (8.18)	5.16 (10.62)	0.807
	IL-12	118.74 (635.16)	20.90 (67.44)	0.26
Th2	IL-4	30.05 (55.26)	34.24 (78.89)	0.73
	IL-5	6.88 (31.65)	4.36 (11.11)	0.575
	IL-13	14.26 (28.41)	20.20 (42.79)	0.359
Th9	IL-9	3.07 (6.13)	3.79 (11.39)	0.652
Th17	IL-17	2.23 (5.46)	8.67 (38.35)	0.176
	IL-21	2.46 (7.57)	5.55 (19.29)	0.229
	IL-22	58.49 (191.83)	152.79 (560.06)	0.197
	IL-23	849.65 (2207.08)	3025.65 (12725.4)	0.17
Chemokine	IL-8	411.87 (1175.18)	912.51 (1851.16)	0.071
	Eotaxin	205.76 (99.13)	208.08 (117.18)	0.905
	MCP-1	786.8 (395.03)	894.20 (575.52)	0.223
NK	IL-15	6.99 (10.90)	6.46 (11.56)	0.796
Regulatory	IL-10	20.89 (50.43)	14.60 (26.08)	0.404

* indicates significant value: * p<0.05.

Interestingly, when patients were subdivided by disease activity and these subgroups were compared individually to HLA-matched and unmatched controls as done in section 3.1, the significant differences found for all patients largely remained (**Supplementary Table 1**), suggesting again that cytokine elevations in patients are independent of disease activity. The only exception was the group of Th17 cytokines which lost most of the significant differences in the subgroup analysis, likely due to the low number of patients expression elevated levels of Th17 cytokines to begin with.

A possible confounding factor is that most of our data points are devised from individual patients, with only a few examples of patients (n=3) with longitudinal data in both active and remittent phases of disease. Of those patients sampled in different disease phases, only one showed considerable cytokine elevation across the board (PV202, **Supplementary Table 2**). For this individual in particular, active disease was generally associated with higher cytokine levels than remission. For the other two patients, general cytokine concentration tended to be lower overall with one patient being higher in active disease and the other in remission (**Supplementary Table 2**).

### 3.4 Therapy does not produce appreciable differences in PV cytokine levels

To evaluate the extent to which therapy influences cytokine levels, we divided patients into 3 groups: 1) off therapy, 2) on minimal therapy, and 3) receiving greater than minimal therapy. No significant differences were found between the various therapy groups when including the entire data set (data not shown). Despite the fact that therapy status does not seem to affect cytokine expression overall in PV patients, we nevertheless directly analyzed cytokine levels in active and remittent PV patients who were *completely off therapy* (n=11, n=27, respectively). Only MCP-1 was found to be significantly elevated in active PV off therapy compared to remittent PV patients off therapy (p-value 0.0298) (**Supplementary Figure 1**). Additionally, when patients were subdivided by therapy and these subgroups were compared individually to HLA-matched and unmatched controls as done in section 3.1, the significant differences found for all patients *vs*. the control group largely remained, again suggesting that cytokine elevations are largely independent of therapy status (**Supplementary Table 3**).

### 3.5 Interleukin 9 and IL-13 distinguish mucocutaneous PV from mucosal PV

**Figure 4:**
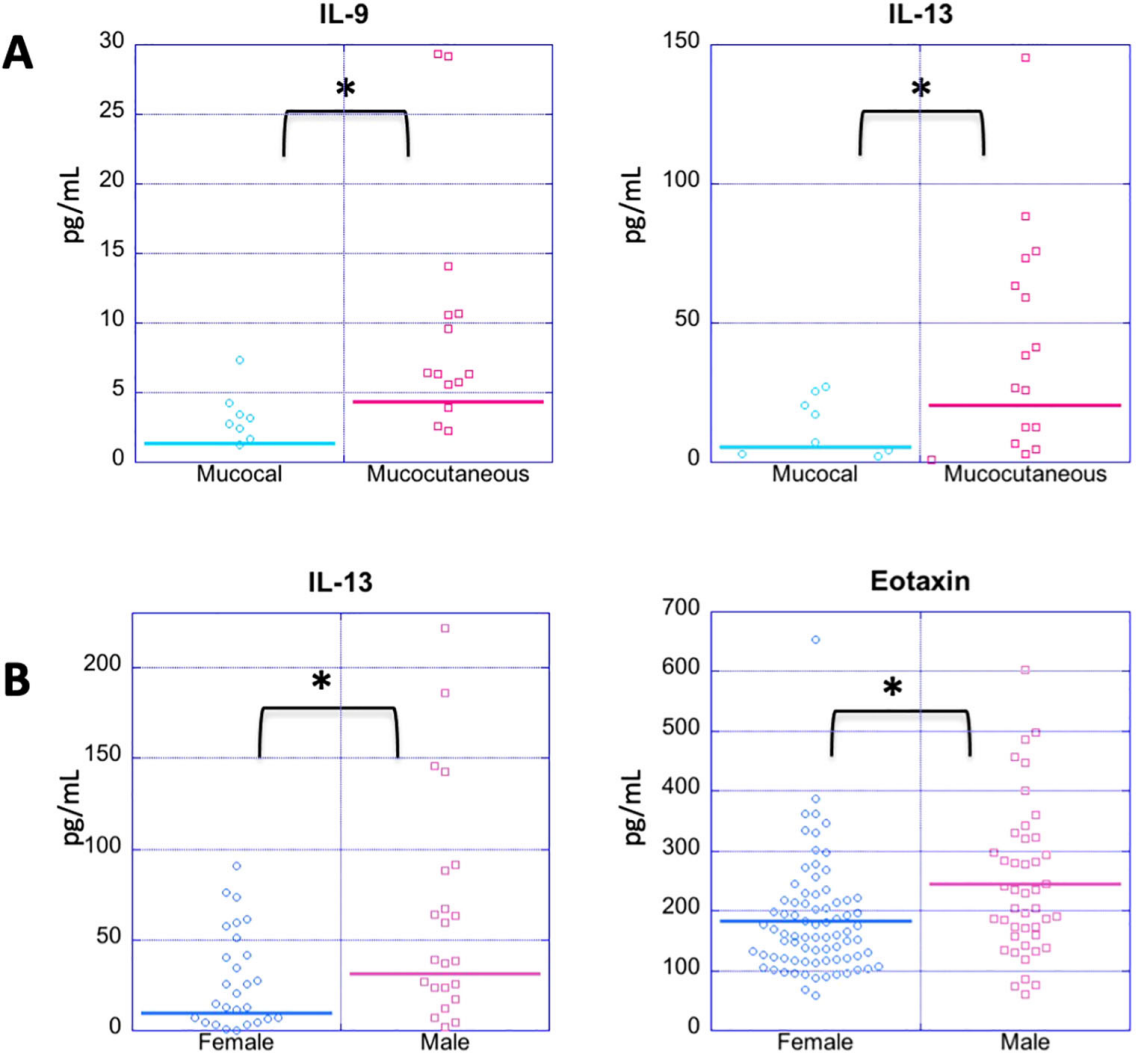
Cytokine expression profiles by disease morphology and gender. **(A)** Dot plots depict cytokine levels in PV patients in patients in active disease with either mucosal or mucocutaneous morphology **(A)** and female *vs*. male PV patients regardless of disease status **(B)**, with each dot representing an individual sample. **(A)** PV patients with active mucocutaneous lesions have significantly elevated levels of IL-9 and IL-13 compared to the patients with active mucosal lesions. **(B)** Males have significantly higher levels of IL-13 and Eotaxin levels than females with PV. Cytokines not showing significant differences for these comparisons are not shown. The horizontal bar in each category represents the mean. P-values ≤ 0.05 are indicated with *.

It has been previously suggested that mucocutaneous PV differs from mucosal PV immunologically (62, 63). In order to assess whether cytokine levels correlate with disease morphology, we analyzed 55 patients that were in active disease, 33 of whom had mucocutaneous lesions, and 20 had mucosal only lesions. Patients with exclusively cutaneous lesions (n=2) were not included in this analysis due to limited numbers. We found the only significant differences between morphological groups to be for Th9 cytokine IL-9 and the Th2 cytokine IL-13, which were significantly elevated in those who had active mucocutaneous PV compared to those who had active mucosal only PV (p-value: 0.035 and 0.022, respectively) (**Figure 4A** and **Table 5**).

**Table 5:** Cytokine/Chemokine levels and group comparison based on disease phenotype.

		Mucosal	Mucocutaneous	p-value
		Mean (SD)	Mean (SD)	
Pro-Inflammatory	IL-1α	123.00 (482.53)	67.79 (152.26)	0.624
	IL-1β	13.26 (31.98)	7.02 (10.32)	0.407
	IL-6	24.22 (86.12)	29.51 (77.98)	0.823
	TNFα	15.06 (15.10)	22.39 (36.36)	0.312
Th1	IFNγ	54.32 (239.31)	91.93 (396.17)	0.703
	IL-2	2.91 (4.88)	6.15 (9.66)	0.111
	IL-12	115.67 (662.03)	66.48 (344.01)	0.677
Th9	IL-9	1.30 (2.00)	4.32 (7.55)	0.035*^3^
Th17	IL-17	4.1 (8.57)	1.06 (1.71)	0.133
	IL-21	3.21 (10.46)	2.02 (5.55)	0.641
	IL-22	67.05 (255.86)	36.27 (102.65)	0.612
	IL-23	666.35 (1958.5)	870.27 (2332.17)	0.734
Th2	IL-4	17.96 (29.78)	39.20 (66.27)	0.117
	IL-5	10.07 (44.23)	5.28 (22.75)	0.656
	IL-13	5.32 (9.20)	20.54 (34.76)	0.022*
Chemokine	IL-8	628.55 (1631.11)	177.21 (534.23)	0.243
	Eotaxin	178.75 (72.22)	209.87 (89.89)	0.172
	MCP-1	867.2 (399.84)	737.27 (402.10)	0.259
NK	IL-15	4.66 (6.93)	8.82 (12.73)	0.13
Regulatory	IL-10	13.09 (20.43)	25.63 (62.99)	0.297

* indicates significant value: * p<0.05.

### 3.6 Interleukin-13 and eotaxin expression distinguish females from males with PV

There is a clear female predominance in PV (64). We analyzed samples from a total of 96 women and 34 men with PV regardless of disease activity. For the most part, we did not observe any significant differences in cytokine/chemokine levels between men and women with PV except for IL-13 and eotaxin (**Figure 4B**). Male patients had higher mean levels of both IL-13 and eotaxin than female patients (male: 31.48 ± 53.08 pg/mL, 244.43 ± 123.55 pg/ml, respectively, and female: 9.91 ± 20.64 pg/mL, 183.79 ± 92.24 pg/ml, respectively) (p-value: 0.0125 and 0.0059, respectively) (**Figure 4B**).

## 4 Discussion

A comprehensive view of the immune mechanisms leading to tissue damage in Pemphigus vulgaris has not been elucidated. Although the presence of B cell-produced autoantibodies directed against specific cell adhesion molecules including Dsg3 and Dsg1 (and perhaps other non-Dsg targets) is critical to lesional development, T cells are certain to play a central role in the pathogenesis of disease. While the rules that govern the coordinated T and B cell response in PV remains unclear, cytokines and chemokines are likely key modulators of the effector and regulatory pathways at play in disease.

It is unlikely that changes in a single cytokine is sufficient to cause the pathological phenomena in any given autoimmune disease (28). Accumulating literature suggests a model in which the action of multiple cytokines ultimately promotes immune-mediated tissue damage (15, 32, 33, 38, 39, 65). A recent study from China found generally elevated levels of Th1, Th2 and Th17 cytokines in PV patients (11). However, it remains clear that a more in-depth knowledge of cytokine profiles in patients is still required for a better understanding of disease mechanisms, and to facilitate the discovery of novel targets for future therapies. To address the mechanisms that underlie the variable and heterogeneous disease expression in PV, we undertook a non-reductionist strategy to comprehensively survey global changes in multiple cytokines/chemokines.

We unexpectedly found that PV patients show a wide variation of cytokine levels across all cytokines tested with levels ranging from undetectable to highly elevated for each individual cytokine regardless of disease phase or therapy status. Interestingly, patients tended to be either ‘high-expressors’ or ‘low-expressors’ of cytokines with ‘high expressors’ exhibiting high serum cytokine concentrations across numerous cytokines (albeit not necessarily all), while ‘low-expressors’ tended to have uniformly lower cytokine levels. Thus, cytokine expression appears to operate relatively, at differing scales dependent on the person. Intriguingly, our data show that if a patient displays elevated cytokine levels, they typically span multiple cytokines within a given pathway (e.g. one or two of the T helper pathways analyzed or generally pro-inflammatory cytokines), but not the entire set of cytokines analyzed. The reasons for this remarkable cytokine pathway heterogeneity among PV patients are intriguing but remain opaque and in need of further exploration, including the investigation of genetic factors such as HLA. Nevertheless, our data have implications for understanding the immunologic basis of clinical heterogeneity, disease operative mechanisms, and have the potential for guiding treatment decisions for patients.

Despite these inter-individual differences, our analysis uncovers a pronounced dysregulation of multiple cytokine immune pathways in PV compared to healthy controls, namely with elevation of the Th2 cytokines IL-4 and IL-5, the Th17 cytokines IL-22 and IL-23, as well as IL-2, IL-9, IL-10 and IL-15. However, a more composite picture emerges when we divide our healthy control population into those controls that carry PV-associated HLA-susceptibility alleles (“HLA-matched”) *vs*. those that do not (“HLA-unmatched”). It is well established that PV-associated HLA alleles such as DRB1*0402 are necessary for T cells to recognize Dsg3 (40), and that Dsg-reactive Th2 cells can be detected in patients with PV (5). Accordingly, PV has been considered a Th2 driven disease (66). Although disease-free, we found that HLA-matched controls express the pro-inflammatory cytokines IL-1α, IL-1β, IL-6, and TNFα, and Th2 cytokine IL-13, and chemokine IL-8 at similar concentrations to that seen in PV patients. These cytokines, however, are significantly higher in PV when compared to HLA-unmatched controls, suggesting that healthy controls carrying the PV associated HLA-haplotypes have a heightened ability to mount a pro-inflammatory and Th2 cytokine response similar to PV patients, while those lacking expression of the PV-associated HLA alleles do not. These findings echo other work in our lab using CyTOF technology to interrogate immune cell distribution in PV patients *vs*. healthy controls. Using CyTOF, we found an increase in Th2 cells in PV patients compared to “HLA-unmatched” controls, while “HLA-matched” controls exhibited similar numbers of Th2 cells to those found in PV patients (*manuscript in preparation*). Our CyTOF studies also showed an increase in Th17 cells in PV patients as compared to HLA-matched controls and HLA-unmatched controls which is supported by our observation of higher levels of the Th17 cytokines IL-21, IL-22 and IL-23 in this study. **Table 1** summarizes differences in cytokine concentration between PV patients and controls that carry PV susceptibility alleles (“HLA-matched”) and those who do not carry HLA susceptibility alleles (“HLA-unmatched”).

Looking more broadly at the genetic basis of autoimmune development, it is striking to note that the pattern of HLA-linked partial activation of autoimmune-linked pathways is not limited only to cytokine expression as revealed in this study, but extends to also gene expression (67), auto-antibody profiles (68), and anti-oxidant status (69) as revealed in published work from our group. These parallel patterns across multiple datasets exploring intersecting biological levels promote a new hypotheses regarding the immunological currents that either promote or prevent autoimmune development (67). Specifically, there may be HLA-independent pathways required for patients to progress to the disease state in PV, but also protective counter-regulatory mechanisms in genetically susceptible (HLA-matched) controls, restraining them from disease despite their genetic susceptibility.

Along these lines, we found that HLA-matched controls had significantly lower levels of IL-10 and IL-15 than PV patients, while HLA-unmatched controls did not, setting up the intriguing hypothesis that the downregulation of these cytokines affords those healthy people that carry genetic susceptibility a certain protection. IL-10, produced by T regulatory cells (amongst others), is thought to be a predominantly inhibitory cytokine. It is known to inhibit production of IFNγ, and activation of macrophages, NK cells, and neutrophils (10, 70). IL-10 plays a critical role in slowing the progression of autoimmune diseases (70). Nevertheless, multiple studies found elevated levels of IL-10 in serum, blister fluid, and supernatants of T cell and B regulatory cell cultures from PV patients compared to controls (25, 40, 45, 71, 72). Also, recent studies suggest that IL-10 is ineffective at accomplishing its downstream regulatory effects in PV (41, 72, 73). Without reaching its downstream target, IL-10 concentration may increase because feedback inhibition does not occur. Perhaps this seemingly contradictory data on IL-10 in PV could be explained by other immune-promoting functions of IL-10, such as induction of B-cell maturation, survival, and differentiation, as well as immunoglobulin production (74–76). Kowalski et al. suggested IL-10 may be higher in PV due this effect on B-cell production of IgG autoantibodies (19). Further, IL-10 can inhibit Th1 cytokines, and thus shift Th1/Th2 balance and promote Th2 cells in the context of PV (25).

Similarly to IL-10, we find that for IL-15, HLA-matched controls show significantly lower serum levels than PV patients, while HLA-unmatched controls do not. IL-15, produced by epithelial cells, fibroblasts, and peripheral blood mononuclear (monocyte enriched) cells promotes immunoglobulin switching, memory immunoglobulins, and NK cell differentiation and proliferation (77, 78). In support of these cytokine data, CyTOF analysis found that the balance of early *vs*. late NK cells was shifted towards late NK cells in PV patients when compared to all controls, as well as when compared to HLA-matched controls (*manuscript in preparation*). Downregulation of IL-15 in matched controls, as shown in this work, could explain why we also see a shift towards early NK cells in these same controls when compared to patients. Also, it is conceivable that a specific downregulation of IL-15 could lead to a reduction in immunoglobulin switching and memory immunoglobulin production and thus mediate disease protection in healthy individuals carrying PV susceptibility alleles.

Surprisingly, we do not see an appreciable difference in cytokine levels between different phases of disease activity, neither in patients regardless of therapy status nor when looking at the subgroup completely off therapy. It is possible that differences between groups in this study may be obscured by the wide variations in cytokine levels across patients. Thus, it will be important in future work to analyze samples taken from patients in a longitudinal study design to truly appreciate if disease activity is paralleled by changed in cytokine levels. In our study, we had three patients with longitudinal samples. Of these three, two exhibited generally higher cytokine levels in active disease, while one had higher levels in remission. Follow up studies are needed to include more patients followed longitudinally across different phases of disease to discern meaningful differences. Similarly, we observed only minimal differences based on disease morphology (mucocutaneous *vs*. mucosal) and based on sex (male *vs*. female). Meaningful differences between these subgroups may again be obscured by the wide variation of cytokine expression between patients.

Taken together, our study reveals that compared to healthy controls (regardless of HLA association), PV patients harbor elevated cytokine expression levels in generally pro-inflammatory- and more specifically Th2 and Th17 pathways, albeit at a very patient-specific, individualized level. This sets up the intriguing future management paradigm that based on an individual patient’s cytokine pattern, cytokine-targeted therapies could be aligned to be maximally effective. Cytokine profiling of individual patients could be leveraged in the future to personalize treatment plans.

Currently, the mainstay of treatment of autoimmune disease remains dependent on potent immunosuppressants that inhibit multiple immune pathways. In 2018, Rituximab was the first biologic to be FDA-approved for treatment of PV. Rituximab is now considered first line for treatment of this disease, although not without side effects. While achieving disease control in a majority of cases, these treatments also place the patient at risk for opportunistic infections and untoward side effects. Front runners of anti-cytokine biologic therapies for various autoimmune diseases include drugs targeting TNFα, IL-1, IL-6, IL-12/IL-23, and IL-17 (79). In the past years, several therapies that target specific cytokines, eg. etanercept (TNFα receptor), adalimumab (anti-TNFα), ustekinumab (anti-IL-23/IL-12), have been developed, with many more in the pipeline. To date, there is limited literature on anti-cytokine therapy in PV. However, multiple recent case reports show Dupilumab, an inhibitor of IL-4 and IL-13 to be effective in treating refractory or severe cases of disease (80–82). Larger studies and clinical trials are necessary to elucidate the true efficacy of dupilumab in PV, as well as explore additional anti-cytokine therapies. Our finding of high cytokine concentrations across one or two T-helper cell pathways may be relevant to predicting which PV patients are likely responders to dupilumab.

While this study emphasizes the importance of cytokines in disease pathogenesis, naturally occurring anti-cytokine autoantibodies are becoming an increasingly prevalent topic in autoimmunity. Though anti-cytokine autoantibodies can be found at low levels in disease-free, healthy subjects, they are found at higher titers in certain autoimmune diseases and immunodeficiencies (83). There are limited reports on the presence of these anti-cytokine autoantibodies in pemphigus and pemphigoid diseases (84, 85). Interestingly, rituximab has been shown to decrease levels of anti-IL-8 autoantibodies in rheumatoid arthritis (86). Given the significance of cytokines in PV pathogenesis, and increasing use of rituximab in PV, further exploration of anti-cytokine autoantibodies is warranted.

Our data support the role of the Th2 pathway in PV, while also lending credence to the increasing literature on the importance of the Th17 pathway. Thus, multiple cytokine pathways appear to be operative in the development of PV. However, in any given patient, not all pathways are simultaneously active; some patients are more Th2 and/orTh17 dominant, while others lean more towards pro-inflammatory or Th1 pathways. We propose a paradigm shift in our understanding of the pathogenesis of PV, specifically that there seem to be different ways individuals can reach disease, either through a majority Th2 and Th17 response, a pro-inflammatory response, or a Th1 response or a combination thereof. The precise molecular means by which patients are directed to different paths leading to PV are unknown, but perhaps there is a genetic basis for which HLA, especially, might play a role. Future mechanistic studies are needed to gain insight into disease risk and prognosis. For now, stratifying patients based on cytokine profiles has the potential to change our approach to therapy. By matching cytokine profiles to more targeted anti-cytokine/anti-pathway treatment, we can envision personalizing care to improve outcomes based on patients’ specific inflammatory profile.

Overall, our studies shed light on the complexities of critical cytokine networks impacting PV on a personalized level. This work can be expected to deepen our understanding of underlying pathogenetic mechanisms relevant to autoimmunity, help to resolve the biologic underpinnings of clinical heterogeneity that confound our ability to prognose and manage patients, and ultimately serve to advance efforts to align appropriately targeted therapies in select patients while reducing side effects associated with current approaches reliant largely on general immunosuppression.

## Data Availability

The raw data supporting the conclusions of this article will be made available by the authors, without undue reservation.
