# Supplementary material for: Cytokine profiling reveals HLA-linked Th2 and Th17 driven immune activation in pemphigus vulgaris patients and genetically susceptible healthy controls
Source: Front Immunol. 2024 Dec 4;15:1500231. doi: 10.3389/fimmu.2024.1500231 (PMC11652493; doi:10.3389/fimmu.2024.1500231)
Supplement: Supplementary file 1 [file Table1.docx]

Supplementary Material

# Supplementary Figures and Tables


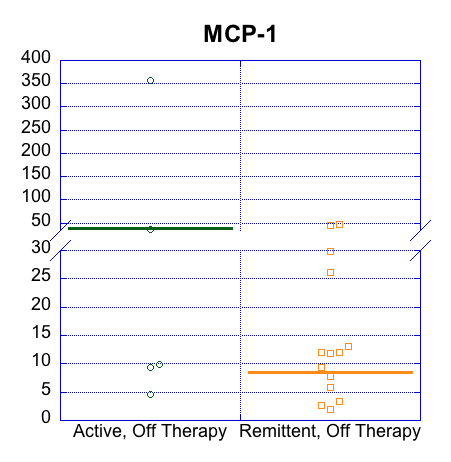


*

**Supplementary Figure 1. Cytokine expression profile by therapy.** Only MCP-1 showed any significant difference with regards to therapy. PV patients who were off therapy and had active disease had significantly higher levels of MCP-1 than patients who were off therapy and in remission. The horizontal bar in each category represents the mean. P-values ≤ 0.05 are indicated with *; p-values ≤ 0.005 are indicated with **.

***

***

**

***

***

***

|  | |  |  |  |  |  |
| --- | --- | --- | --- | --- | --- | --- |
|  |  | **PV Active vs. HLA-Matched Controls** | | **PV Active**  **vs. HLA-Unmatched Controls** | **PV Remission**  **vs. HLA-Matched Controls** | **PV Remission**  **vs. HLA-Unmatched Controls** |
| **Pro - Inflammatory** | **IL-1α** | 0.2714 | | **0.0603(*)** | 0.5263 | **0.0227*** |
|  | **IL-1β** | 0.3480 | | **0.0326*** | 0.8780 | **0.0120*** |
|  | **IL-6** | 0.2940 | | **0.0177*** | 0.8985 | **0.0042**** |
|  | **TNF-α** | 0.3086 | | **0.0126*** | 0.6993 | **0.0002***** |
|  |  |  | |  |  |  |
| **Th1** | **IFN-γ** | 0.1572 | | 0.1113 | 0.8796 | 0.3880 |
|  | **IL-2** | **0.0120*** | | **0.0903(*)** | **0.0079**** | **0.0633(*)** |
|  | **IL-12** | 0.1900 | | 0.1547 | 0.9624 | 0.0345* |
|  |  |  | |  |  |  |
| **Th2** | **IL-4** | 0.1347 | | 0.1753 | 0.1176 | 0.1517 |
|  | **IL-5** | 0.1704 | | **0.0962(*)** | **0.0678(*)** | **0.0023**** |
|  | **IL-13** | 0.7701 | | 0.1599 | 0.9638 | **0.0514(*)** |
|  |  |  | |  |  |  |
| **Th9** | **IL-9** | **0.0060**** | | **0.0280*** | **0.0179*** | **0.0427*** |
|  |  |  | |  |  |  |
| **Th17** | **IL-17** | 0.6697 | | 0.4690 | 0.9532 | 0.3443 |
|  | **IL-21** | 0.6507 | | **0.0599(*)** | 0.4951 | **0.0563(*)** |
|  | **IL-22** | 0.1582 | | **0.0796(*)** | 0.1449 | **0.0579(*)** |
|  | **IL-23** | 0.1822 | | 0.1254 | 0.1626 | 0.0972 |
|  |  |  | |  |  |  |
| **Chemokine** | **IL-8** | 0.3497 | | 0.1025 | 0.7306 | **0.0015**** |
|  | **Eotaxin** | 0.0494* | | 0.3843 | 0.1418 | 0.7935 |
|  | **MCP-1** | 0.5885 | | 0.8158 | 0.8735 | 0.4188 |
|  |  |  | |  |  |  |
| **NK** | **IL-15** | **0.0068**** | | 0.0946(*) | **0.0080**** | 0.1244 |
|  |  |  | |  |  |  |
| **Regulatory** | **IL-10** | **0.0197*** | | 0.3153 | **0.0018**** | 0.6109 |

**Supplementary Table 1**. Results of heteroscedastic T-tests comparing patients subdivided by activity level compared to HLA-Matched and HLA-Unmatched controls. (*) p<0.1, * p<0.05, **p<0.01,***p<0.001. Bolded results correspond to our data on patients not subdivided by disease activity.

|  | **cytokine** | **IL-17A** | **IL-22** | **IL-21** | **IL-23** | **IL-2** | **IL-12P70** | **IFNγ** | **IL-4** | **IL-5** | **IL-13** | **IL-1α** | **IL-1β** | **IL-6** | **IL-8** | **TNFα** | **Eotaxin** | **IL-9** | **IL-10** | **IL-15** | **MCP-1** |
| --- | --- | --- | --- | --- | --- | --- | --- | --- | --- | --- | --- | --- | --- | --- | --- | --- | --- | --- | --- | --- | --- |
|  | **mean** | 5.55 | 105.31 | 4.13 | 1972.93 | 4.95 | 61.53 | 63.01 | 32.52 | 5.26 | 17.51 | 76.6 | 20.75 | 86.49 | 655.03 | 30.32 | 201.74 | 3.43 | 17.32 | 6.65 | 841.06 |
| **PVID** | **disease activity** |  |  |  |  |  |  |  |  |  |  |  |  |  |  |  |  |  |  |  |  |
| PV202 | A | 0 | *293* | *24.65* | *4685* | *26.3* | *1950.0* | *1380* | *164.5* | *128* | *73.35* | *735* | *22.85* | *277* | *326.5* | *155* | *149.0* | *29.35* | *356* | *27.35* | *395.5* |
| PV202 | NR | 2.25 | 0 | 6.905 | 1147.5 | 9.505 | 0.0 | 7.99 | 81.6 | 1.82 | 34.65 | 89.25 | 8.78 | 8.305 | 41.8 | 9.395 | 58.6 | 3.655 | 29.6 | 11.8 | 236.5 |
| PV202 | NR | 0 | 0 | 11.55 | 1475 | 11.45 | 16.7 | 60 | 103.85 | 3 | 40.3 | 129.5 | 10.43 | 23.45 | 54.5 | 11.9 | 67.6 | 4.27 | 34.9 | 14.5 | 367.5 |
| PV202 | LTR | 5.395 | 54.9 | 10.07 | 1400 | 8.15 | 21.1 | 94.75 | 81.2 | 1.7 | 23.2 | 67.8 | 7.545 | 11.6 | 246.5 | 12 | 74.6 | 2.82 | 27.3 | 9.85 | 341 |
| PV202 | NR | 2.54 | 0 | 8.12 | 1250 | 7.33 | 0.0 | 14.45 | 63.8 | 1.4 | 27.55 | 72.4 | 6.995 | 18.6 | 36.5 | 18.2 | 75.0 | 2.73 | 24.6 | 8.875 | 308.5 |
| PV242 | A | 0.6725 | 0 | 25.25 | 111.15 | 23.95 | 53.4 | 9.405 | 16.5 | 4.465 | 41.4 | 0 | 35.6 | 23.8 | 57.75 | 28.1 | 182 | 5.75 | 16.7 | 39.8 | 721.5 |
| PV242 | LTR | *3.515* | *93.4* | *1255* | *7930* | *56.65* | *105.0* | *43.55* | *87.05* | *17.8* | *57.75* | *232* | *64.3* | *43.2* | 20.5 | *45.1* | *141* | *16.85* | *33.8* | *56* | 541.5 |
| PV247 | A | 0 | 0 | 0 | 0 | *11.8* | 0.7 | *6.63* | *79.7* | 0.5705 | *25.4* | *82.15* | *12.25* | *8.855* | 24.1 | 4 | *235.5* | 3.43 | *25* | *19.1* | *1870* |
| PV247 | NR | 0 | 0 | 0 | 0 | 0 | 0.0 | 0 | 0 | 0 | 0 | 0 | 0 | 0 | 35.75 | 4.435 | 145 | 0 | 0 | 0 | 784 |

**Supplementary Table 2.** Cytokine concentrations (pg/mL) for one PV patient during multiple different disease states. A = Active, NR = newly remittent (2-5 months remission), LTR = long term remittent (>6 months remission). The second row of this table indicates the mean cytokine concentration for all of the patients analyzed in this study. In active disease, this individual has generally high cytokine levels, especially in the Th2 pathway, when compared to the mean cytokine concentration for all of the patients in the study. When looking at differences between disease states for this individual, we see this patient has generally higher cytokine levels in active disease when compared to newly remittent and long term remission. Italics indicate the generally higher values for the active or remittent state depending on the patient

|  | |  |  | |  | |  | |  | | |  | |
| --- | --- | --- | --- | --- | --- | --- | --- | --- | --- | --- | --- | --- | --- |
|  |  | **PV Off therapy vs. HLA-Matched Controls** | | **PV Off Therapy**  **vs. HLA-Unmatched Controls** | | **PV Min Therapy**  **vs. HLA-Matched Controls** | | **PV Min Therapy**  **vs. HLA-Unmatched Controls** | | **PV >Min Therapy**  **vs. HLA-Matched Controls** | **>PV Min Therapy**  **vs. HLA-Unmatched Controls** | |  |
| **Pro - Inflammatory** | **IL-1α** | 0.2441 | | **0.0864(*)** | | 0.3116 | | **0.0349*** | | 0.9100 | 0.1425 | |  |
|  | **IL-1β** | 0.4635 | | **0.0299*** | | 0.3552 | | **0.0199*** | | 0.3802 | **0.0308*** | |  |
|  | **IL-6** | 0.5185 | | **0.0155*** | | 0.1915 | | **0.0067**** | | 0.5217 | **0.0171*** | |  |
|  | **TNF-α** | 0.9026 | | **0.0007***** | | 0.1545 | | 0.3515 | | 0.4592 | **0.0054**** | |  |
|  |  |  | |  | |  | |  | |  |  | |  |
| **Th1** | **IFN-γ** | 0.1288 | | 0.0995(*) | | 0.9173 | | 0.3664 | | 0.3728 | 0.8413 | |  |
|  | **IL-2** | **0.0426*** | | 0.2257 | | **0.0044**** | | **0.0130*** | | **0.0906(*)** | 0.3691 | |  |
|  | **IL-12** | 0.1817 | | 0.1571 | | 0.9950 | | 0.0492* | | 0.9209 | 0.1478 | |  |
|  |  |  | |  | |  | |  | |  |  | |  |
| **Th2** | **IL-4** | 0.3866 | | 0.4727 | | 0.0488* | | 0.0596(*) | | 0.2548 | 0.3092 | |  |
|  | **IL-5** | 0.1251 | | **0.0818(*)** | | 0.2372 | | **0.0543(*)** | | 0.3090 | **0.0315*** | |  |
|  | **IL-13** | 0.6983 | | 0.2336 | | 0.6277 | | **0.0370*** | | 0.7860 | 0.2102 | |  |
|  |  |  | |  | |  | |  | |  |  | |  |
| **Th9** | **IL-9** | **0.0306*** | | **0.0969(*)** | | **0.0071**** | | **0.0189*** | | **0.0612(*)** | 0.1040 | |  |
|  |  |  | |  | |  | |  | |  |  | |  |
| **Th17** | **IL-17** | 0.4870 | | 0.2260 | | 0.5008 | | 0.7131 | | 0.2860 | 0.9825 | |  |
|  | **IL-21** | 0.6480 | | 0.1005 | | 0.3126 | | 0.1188 | | 0.7677 | **0.0806(*)** | |  |
|  | **IL-22** | 0.2523 | | 0.1615 | | 0.2498 | | 0.1774 | | 0.1697 | **0.0428*** | |  |
|  | **IL-23** | 0.2586 | | 0.2034 | | 0.2607 | | 0.2133 | | 0.1589 | **0.0496*** | |  |
|  |  |  | |  | |  | |  | |  |  | |  |
| **Chemokine** | **IL-8** | 0.8993 | | **0.0084**** | | 0.2675 | | 0.4438 | | 0.5478 | **0.0193*** | |  |
|  | **Eotaxin** | 0.2696 | | 0.9379 | | 0.1874 | | 0.9194 | | 0.0369* | 0.2789 | |  |
|  | **MCP-1** | 0.6038 | | 0.7978 | | 0.7798 | | 0.6034 | | 0.8541 | 0.4760 | |  |
|  |  |  | |  | |  | |  | |  |  | |  |
| **NK** | **IL-15** | **0.0553(*)** | | 0.3452 | | **0.0035**** | | 0.0174* | | **0.0445*** | 0.3393 | |  |
|  |  |  | |  | |  | |  | |  |  | |  |
| **Regulatory** | **IL-10** | 0.1031 | | 0.4800 | | **0.0042**** | | 0.3102 | | **0.0051**** | 0.6272 | |  |

**Supplementary Table 3**. Results of heteroscedastic T-tests comparing patient subdivided by therapy level compared to HLA-Matched and HLA-Unmatched controls. (*) p<0.1, * p<0.05, **p<0.01,***p<0.001. Bolded results correspond to our data on patients not subdivided by therapy level.
